# Isolation of avian influenza H5N1 virus from vaccinated commercial layer flock in Egypt

**DOI:** 10.1186/1743-422X-9-294

**Published:** 2012-11-27

**Authors:** Elham F El-Zoghby, Abdel-Satar Arafa, Walid H Kilany, Mona M Aly, Hafez M Hafez

**Affiliations:** 1 National Laboratory for Veterinary Quality Control on Poultry Production, Animal Health Research Institute, P.O. Box 246-Dokki, Giza, 12618, Egypt; 2 Institute of Poultry Diseases, Free University of Berlin, Koenigsweg 63, Berlin, 14163, Germany; 3 Federal Research Institute for Animal Health, Friedrich Loeffler Institute, Institute of Molecular Biology, Suedufer 10, Greifswald, 17493, Germany

**Keywords:** Highly pathogenic avian influenza, H5N1, Egypt, Vaccination failure, Backyards, Live bird markets

## Abstract

**Background:**

Uninterrupted transmission of highly pathogenic avian influenza virus (HPAIV) H5N1 of clade 2.2.1 in Egypt since 2006 resulted in establishment of two main genetic clusters. The 2.2.1/C group where all recent human and majority of backyard origin viruses clustered together, meanwhile the majority of viruses derived from vaccinated poultry in commercial farms grouped in 2.2.1.1 clade.

**Findings:**

In the present investigation, an HPAIV H5N1 was isolated from twenty weeks old layers chickens that were vaccinated with a homologous H5N1 vaccine at 1, 7 and 16 weeks old. At twenty weeks of age, birds showed cyanosis of comb and wattle, decrease in egg production and up to 27% mortality. Examined serum samples showed low antibody titer in HI test (Log_2_ 3.2± 4.2). The hemagglutinin (HA) and neuraminidase (NA) genes of the isolated virus were closely related to viruses in 2.2.1/C group isolated from poultry in live bird market (LBM) and backyards or from infected people. Conspicuous mutations in the HA and NA genes including a deletion within the receptor binding domain in the HA globular head region were observed.

**Conclusions:**

Despite repeated vaccination of layer chickens using a homologous H5N1 vaccine, infection with HPAIV H5N1 resulted in significant morbidity and mortality. In endemic countries like Egypt, rigorous control measures including enforcement of biosecurity, culling of infected birds and constant update of vaccine virus strains are highly required to prevent circulation of HPAIV H5N1 between backyard birds, commercial poultry, LBM and humans.

## Findings

### Background

A devastating highly pathogenic avian influenza virus (HPAIV) of H5N1 subtype first emerged in poultry and further transmitted to human in Hong Kong in 1997 [1]. The spread of the H5N1 virus to Europe and Africa was due to a large-scale outbreak of H5N1 infection that occurred in migratory waterfowl in Qinghai Lake (China) in 2005 [2]. To date, ten different genetic clades (0 – 9) of H5N1 virus have been distinguished which further diversified into subclades [3]. The HPAIV H5N1 of clade 2.2.1 has been introduced into Egypt in early 2006 [4] probably via infected wild ducks [5]. Despite control efforts, the virus had become endemic in poultry in Egypt since 2008 [6]. Egypt embarked mainly on inactivated H5N1 and H5N2 vaccines to limit the spread of H5N1 virus and minimize its socioeconomic impacts [7]. However, circulation of the HPAIV H5N1 in different poultry species (chickens, ducks, turkeys, etc.), in addition to donkeys and possibly pigs was reported [8-10]. Moreover, out of 168 infected human in Egypt, 60 cases were fatal until July 27, 2012 [11].

In Egypt, H5N1 isolated from humans and a large group of viruses isolated from backyard ducks and chickens clustered in a distinct genetic group designated “2.2.1/C” [12], meanwhile, majority of viruses derived from vaccinated poultry in commercial farms were found in newly named 2.2.1.1 clade [3,13]. A virus belonging to 2.2.1/C group has been also isolated from donkeys in Egypt [8]. Those 2.2.1/C viruses harbor conspicuous mutations in the HA and NA proteins, were thought to be responsible for decreasing virulence in mammals as a step towards adaptation to the human population [14,15]. On the contrary, viruses cluster in clade 2.2.1.1, also known as variant 2.2.1 viruses, had extensive amino acids substitutions in or adjacent to the immunogenic epitopes at the proximal globular head region of the HA protein which could enable continuous circulation of the virus in and among commercial poultry despite large scale vaccination campaigns [16,17]. Regular nationwide active, passive and targeted surveillance revealed that HPAV H5N1 is perpetuated in many commercial farms, backyards and live bird markets (LBM) [4,7,9,16,18]. Culling of infected birds occurs infrequently in Egypt [6].

Backyard birds and commercial poultry are kept in very close contact with humans due to integration of both farms and houses in the same buildings. Employees in commercial farms usually maintain their own household birds. Furthermore, selling of remaining feed, utensils and equipment from commercial farms to the rural family poultry often occurs in Egypt. Backyard chickens, ducks and geese are mostly reared together and roam freely in the vicinity of the house in close contact with human, particularly children. More than 70% of the Egyptian poultry production from commercial or backyard sectors is marketed through LBM [6]. All H5N1 infected human cases, except three cases, were linked to direct contact of human with sick or apparently healthy birds in backyards and/or LBM [19].

Here we describe the isolation and molecular characterization of an HPAI H5N1 virus isolated from twenty weeks old chicken layer flock, which was vaccinated three times with a commercial inactivated H5N1 vaccine.

### Methodology

A commercial chicken farm with 23,699 Hisex-brown layers kept in cages; chickens were vaccinated three times at weeks 1, 7, and 16 with a commercial inactivated H5N1 vaccine seeded by A/Goose/Guangdong/1/1996/H5N1 (Re-1 YEBIO, Harbin, China). Vaccination of birds was conducted by the owner as part of usual practice. Ten tracheal swabs and ten cloacal swabs were collected randomly at day 7 after onset of clinical signs. At the same time, ten serum samples were collected for further laboratory investigation. Ten swabs were pooled according to Manual of Diagnostic Tests and Vaccines for Terrestrial Animals [20]. No experimental research was conducted in this study and the birds were handled according to the standard guidelines [20]. Samples were collected during the routine nationwide influenza surveillance program after the ministerial decree number 221/2006 in charge the National Laboratory for Veterinary Quality Control on Poultry Production (NLQP) for official diagnosis and surveillance of AIV in Egypt.

Virus isolation trials were carried out in 10-day-old SPF hatching eggs via allantoic sac inoculation [20]. The virus titer was estimated by mean egg-infective dose (EID_50_/0.1mL) according to Reed and Muench [21]. For detection of avian influenza H5–specific antibodies in the serum samples; hemagglutination inhibition (HI) test was performed in V-bottom; 96-well microtiter plates with four hemagglutinating units (4HAU) of the indicated homologous A/Goose/Guangdong/1/1996 H5N1 antigen, supplied by the vaccine-producing company (YEBIO, Harbin, China) and 1% of chicken erythrocytes according to the standard protocol [20]. Results were interpreted as the reciprocal of the last well that showed complete inhibition of the hemagglutination activity of the used H5 antigen.

Viral RNA from fluid of pooled swabs was extracted using a MagNA Pure LC Total Nucleic Acid Extraction kit following manufacturer’s instructions and MagNA Pure LC instrument (Roche, Mannheim, Germany). The RT-qPCR reaction was done using one step Real-Time PCR Kit (Qiagen, Valencia, CA.) as recommended by the manufacturer. Partial matrix (M) gene segment of AIV from viral RNA was amplified according to Spackman et al. [22] using forward primer 5^′^-AGA TGA GTC TTC TAA CCG AGG TCG-3^′^, reverse primer 5^′^-TGC AAA AAC ATC TTC AAG TCT CTG-3′and probe 5^′^ FAM-TCA GGC CCC CTC AAA GCC GA-TAMRA-3^′^. The RT-qPCR reaction was done in Stratagene MX3005P real time PCR machine (Stratagene, Agilent Technologies, Santa Clara, CA). Thereafter, H5 and N1 genes were amplified using generic avian influenza virus H5N1 Real Time RT-PCR RT3 Kits (Roche, Mannheim, Germany) according to the manufacturer guidelines in LightCycler® 2.0 machine.

**Figure 1:**
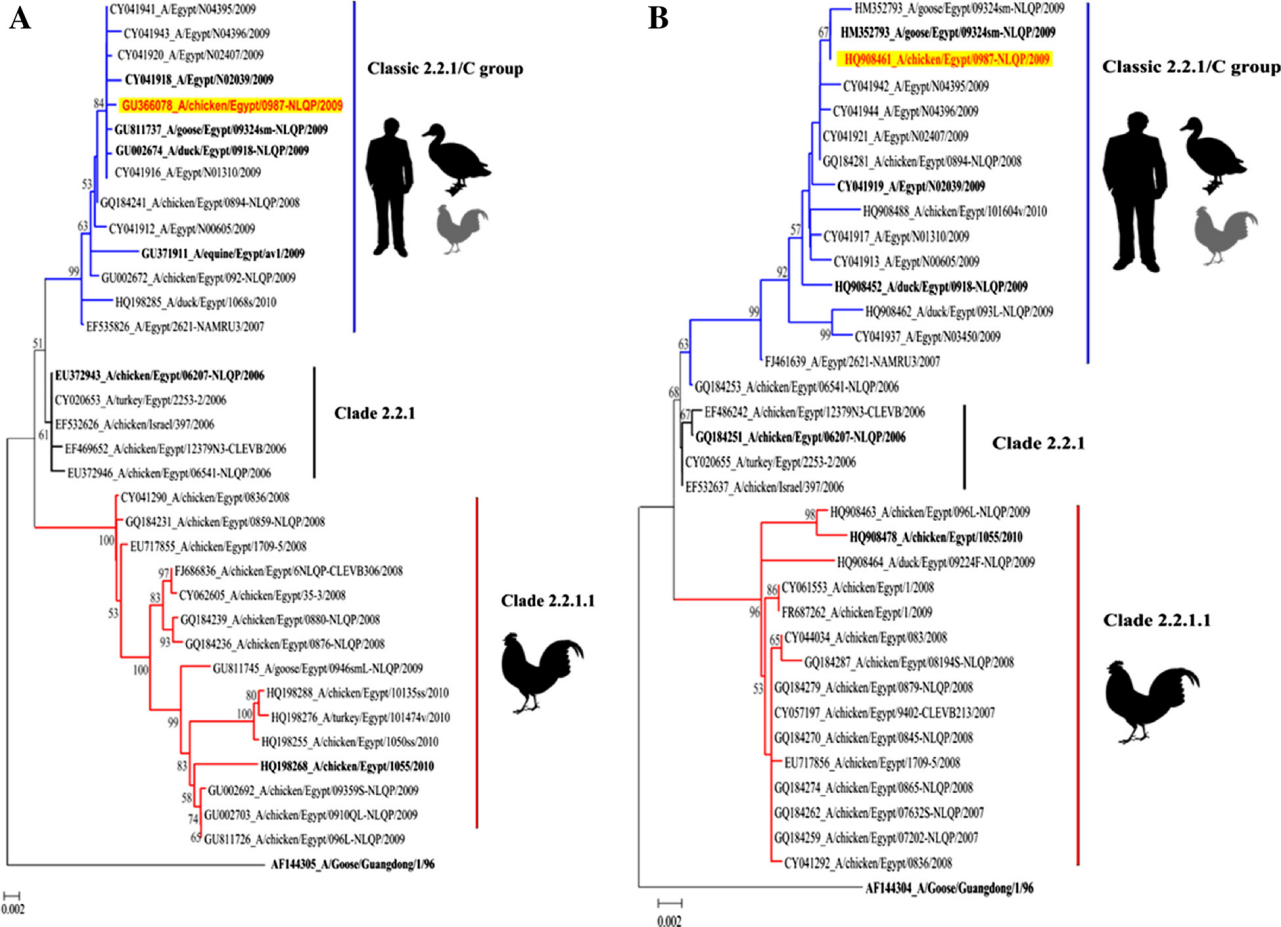
**Phylogenetic trees of H5 (A) and N1 (B) genes of A/chicken /Egypt/0987-NLQP/2009 virus with other H5N1 viruses currently circulating in poultry and human in Egypt.** The evolutionary history of the HA and NA was inferred using the Neighbor-Joining method with 1000 bootstrap replicates and the evolutionary distances were computed using the Maximum Composite Likelihood method implemented in MEGA5. The bootstrap values are shown next to the branches. Trees were edited for publication using Inkscape software. Virus obtained from this study is highlighted in yellow while other related viruses are written in bold. The virus clustered with H5N1 viruses isolated from poultry in backyards, live bird market and confirmed human infections. Two major H5N1 genetic clusters are currently co-circulating in Egypt: newly designated 2.2.1.1 clade (depicted in red) representing variant viruses isolated exclusively from vaccinated poultry in commercial farms and group C of 2.2.1 clade (blue) denoted 2.2.1/C group that mainly isolated from human and backyard birds according to the recent classification of WHO/OIE/FAO Evolution Working Group. The 2.2.1 index virus introduced into 2006 (black) was no longer isolated and was denoted as 2.2.1 clade.

Amplification of the open reading frame of the HA and NA gene segments was conducted as previously described [16]. The full coding sequences of the HA and NA genes of the isolated virus were conducted using BigDye Terminator v3.1 Cycle Sequencing Kit on an automatic sequencer (ABI-3130; Applied Biosystems, Foster City, CA). The produced sequences were aligned with BioEdit version 7.0.9.0 [23]. Amino acid sequence was deduced and a BLASTN search was performed to identify the query sequence and to find similar sequences. Phylogenetic trees of the obtained H5 and N1 genes and other relevant H5N1 genes retrieved from the GenBank data base were generated using the neighbor-joining method with 1000 bootstrap replicates and the evolutionary distances were computed using the Maximum Composite Likelihood method implemented in MEGA5 [24]. Trees were edited for publication using Inkscape software 0.48.1 as shown in Figure 1. Prediction of N-linked glycosylation sites was done by the NetNGlyc 1.0 Server that examines the sequence context of N–X–S/T sequons [25].

The virus isolated in this study was designated as A/chicken/Egypt/0987-NLQP/2009(H5N1) and referred to as “Layer/0987”. Identity matrix of the isolated virus with other six H5N1 viruses was done: (1) A/goose/Egypt/09324sm-NLQP/2009 [GenBank: GU811737 and HM352793 for the HA and NA genes, respectively] isolated from geese in live bird market (LBM) in the same region and referred to as LBM/Gs. (2) A/duck/Egypt/0918-NLQP/2009 [GenBank: GU002674 and HQ908452] isolated from backyard birds referred to as BY/Dk. (3) A/Egypt/N02039/2009 [GenBank: CY041918 and CY041919] was detected from 32 months old boy few days before the incidence of the current outbreak in commercial-layer flock from the same village (case number 57 in the WHO [26]) and referred to as Human/N02039. Those three viruses belong to the 2.2.1/C group. (4) A/chicken/Egypt/06207-NLQP/2006 [GenBank: EU372943 and GQ184251] was isolated in February 2006 and referred to as Index/2006. (5) A/chicken/Egypt/1055/2010 [GenBank: HQ198268 and HQ908478] a representative virus of the 2.2.1.1 variant clade that was isolated from vaccinated chickens and referred to as Variant/1055. (6) Layer chickens in this study were vaccinated by a commercial vaccine modified by reverse genetic where monobasic-HA and NA genes originated from H5N1 A/Goose/Guangdong/1/96 [GenBank: AF144305 and AF144304] referred to as GsGd/96. The latter was considered to be the parent virus of clade 2.2 [27]. Numbering of amino acid residues of the HA (H5 numbering after removal of the signal peptide) and NA proteins were done in comparison to GsGd/96 as standard (N1 numbering).

Tertiary structures of H5 and N1 glycoprotein monomers were generated as PDB using 3D-JIGSAW from Index/2006 virus [28]. Location of amino acid substitutions was imposed on the HA and NA proteins using RasTop software version 2.7.1 [29] and further edited by Inkscape.

### Results

At twenty weeks of age, some birds showed cyanosis of comb and wattles, hemorrhages on the shank and a total mortality estimated to be 27%. In addition, there was a drop in egg production (20%) and an increase of the number of misshaped eggs (depigmentation, soft eggshell, shell-less and rough eggs). The course of the disease took more than 21 days from onset of clinical illness until depopulation of the flock.

After inoculation of embryonated chicken eggs with tracheal and cloacal swabs media, all embryos died within 48 hours post inoculation. Hemagglutination activity in the harvested allantoin fluid was determined. The isolated virus was confirmed to be an H5N1 virus by partial amplifications of the M, HA and NA genes of avian influenza viruses using specific real-time reverse transcription– polymerase chain reaction (RT-qPCR). The median egg infectious dose was 7.2 EID_50_/0.1mL. Serum samples collected at 7 days after the onset of clinical signs had mean HI titer of 3.2 log_2_ (standard deviation ± 4.3 log_2_) using homologous A/Goose/Guangdong/1/1996 H5N1 antigen, supplied by the vaccine-producing company (YEBIO, Harbin, China).

The full coding H5 and N1 gene sequences generated in this study were submitted to GenBank under accession numbers GU366078 and HQ908461 for the HA and NA genes, respectively. A/chicken/Egypt/0987-NLQP/2009(H5N1) isolated in this study had 99.76% and 99.65% HA and 99.85% and 99.78% NA nucleotides and amino acids identity, respectively with an H5N1 virus isolated from LBM from the same village as shown in Table 1. A/chicken/Egypt/0987-NLQP/2009 had 99.82% and 99.65% HA and 99.64% and 99.49% NA nucleotides and amino acids identity, respectively with an H5N1 virus isolated from backyard birds in addition to 99.77% and 99.47% HA and 99.55% and 99.55% NA nucleotides and amino acids identity, respectively with an H5N1 virus isolated from 32 months old boy from the same village. However, the virus isolated in this study had nucleotides and amino acids identities of 94.28% and 94.53% for the HA and 93.1% and 92.04% for the NA with the vaccine strain (GsGd/96), respectively as shown in (Table 1). Both HA and NA genes clustered with 2.2.1/C group but not with viruses isolated from vaccinated commercial poultry in 2.2.1.1 clade (Figure 1).

**Table 1:** Percentage identity of the hemagglutinin and neuraminidase sequences of A/chicken/Egypt/0987-NLQP/2009 in relation to other Egyptian H5N1 viruses and vaccine strain

**Hemagglutinin**	^**1**^**Layer/0987**	^**2**^**LBM/Gs**	^**3**^**BY/Dk**	^**4**^**Human/N02039**	^**5**^**Index/2006**	^**6**^**Variant/1055**	^**7**^**GsGd/96**
**Layer/0987**		99.77	99.83	99.77	98.90	96.19	94.28
**LBM/Gs**	99.65		99.94	99.88	98.96	96.19	94.46
**BY/Dk**	99.65	100.00		99.94	99.02	96.13	94.52
**Human/N02039**	99.47	99.82	99.82		98.96	96.25	94.46
**Index/2006**	98.94	99.29	99.29	99.12		96.88	96.02
**Variant/1055**	94.89	95.24	95.24	95.06	95.59		92.96
**GsGd/96**	94.53	93.47	94.71	94.53	95.41	91.71	
**Neuraminidase**	**Layer/0987**	**LBM/Gs**	**BY/Dk**	**Human/N02039**	**Index/2006**	**Variant/1055**	**GsGd/96**
**Layer/0987**		99.85	99.64	99.55	98.58	97.30	93.01
**LBM/Gs**	99.78		99.75	99.78	98.73	97.43	91.54
**BY/Dk**	99.49	99.58		99.49	98.56	97.20	90.59
**Human/N02039**	99.55	99.70	99.41		98.74	97.43	91.63
**Index/2006**	98.88	99.10	98.98	98.89		98.49	92.62
**Variant/1055**	97.96	98.19	97.96	97.96	99.09		91.76
**GsGd/96**	92.04	92.26	91.35	92.04	93.18	92.19	

Percentage identity of nucleotide (above the diagonal) and amino acid (below the diagonal).

^1^ The virus isolated in this study was designated as A/chicken/Egypt/0987-NLQP/2009 under GenBank accession numbers GU366078 and HQ908461 for the HA and NA genes, respectively.

^2^ A/goose/Egypt/09324sm-NLQP/2009 [GenBank: GU811737 and HM352793] isolated from geese in live bird market (LBM) in the same region.

^3^ A/duck/Egypt/0918-NLQP/2009 [GenBank: GU002674 and HQ908452] isolated from backyard birds.

^4^ A/Egypt/N02039/2009 [GenBank: CY041918 and CY041919] isolated from 32 months old boy from the same village.

^5^ A/chicken/Egypt/06207-NLQP/2006 [GenBank: EU372943 and GQ184251] was isolated in February 2006 and represents the index 2.2.1 Egyptian viruses.

^6^A/chicken/Egypt/1055/2010 [GenBank: HQ198268 and HQ908478] variant 2.2.1.1 virus isolated from vaccinated chickens in a commercial farm.

^7^ A/Goose/Guangdong/1/96 [GenBank: AF144305 and AF144304] vaccine strain used in vaccination of the current commercial layer flock.

**Figure 2:**
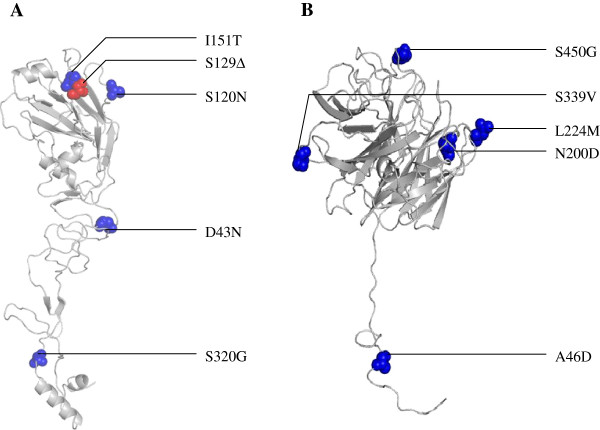
**Predicted location of amino acid substitutions found in the isolated virus on the tertiary structure of HA H5 (A) and N1 NA (B) monomers.** Shown are the substitutions in D43N, S120N, I151T and S320G in the HA and A46D, N200D, L224M, S339V and S450G in the NA proteins. Substitutions have been imposed upon the tertiary structure of A/chicken/Egypt/06207-NLQP/2006(H5N1) (index virus of clade 2.2.1 introduced into Egypt in early 2006). Protein modeling was generated by JIGSAW [28] and edited by RasTop version 2.7.1 and further edited by Inkscape.

The proteolytic cleavage site of the HA had polybasic amino acid motif “ERRRKKR/GLF”, typical for HPAIV of clade 2.2. A deletion within the receptor binding site at position 129S (H5 numbering) and additional four amino acid substitutions namely; D43N, S120N, I151T and S320G of viral H5 protein in comparison to H5N1 virus introduced into Egypt in 2006 (Index/2006) were found. All mutations located in the HA1 on the globular head domain (Figure 2). In addition, 14 synonymous mutations were also found. Seven potential glycosylation sites (residues 11, 23, 165, 193, 286, 484 and 543) in the HA protein were predicted. De-glycosylation (loss of asparagine and existence of aspartic acid instead) of amino acid residue 154 was observed.

The NA of A/chicken/Egypt/0987-NLQP/2009 has a deletion of 20 amino acids in the stalk region (49 – 68; N1 numbering) in comparison to GsGd/96. Furthermore, in comparison to H5N1 virus introduced into Egypt in 2006, A/chicken/Egypt/0987-NLQP/2009 had five amino acid substitutions in the NA protein namely; A46D, N200D, L224M, S339V, and S450G and 14 synonymous mutations in the NA gene. There was no molecular marker of neuraminidase inhibitor resistance. The NA protein had three predicted potential glycosylation sites; residues 68, 126 and 215. On the contrary, GsGd/96 virus had additional four potential glycosylation sites that located in the deleted stalk region of A/chicken/Egypt/0987-NLQP/2009.

### Discussion

Twenty weeks old layer chickens flock vaccinated three times with inactivated H5N1 vaccine suffered from respiratory distress and some birds had signs commonly seen in poultry infected with HPAIV. HPAIV H5N1 was isolated from tracheal and cloacal swabs media collected after onset of clinical signs during regular national surveillance. Full H5 and N1 coding gene sequences showed a close genetic relationship between the obtained virus and viruses of LBM, backyards and human origins. Due to insufficiency of epidemiological data, the source of this infection through either LBM, backyard birds or from humans cannot be deduced.

Despite repeated vaccination using a homologous H5N1 vaccine, the chickens exhibited observable distress and mortality. This suggests updated poultry vaccine may be necessary in endemic areas like Egypt. Tian et al., [30] found that the HI titer of > 4log_2_ indicated sufficient protection of vaccinated chickens against HPAIV H5N1 infection. In this study, anti-H5 antibody titer was low with variable individual titers (3.2 ± 4.3 log_2_) after three successive vaccinations of Hisex brown-layers. Recent experimental studies showed that specific-pathogen-free (SPF) chickens vaccinated with this inactivated H5N1 vaccine evoked high HI titer (7.0 ± 0.8 log_2_) and were protected against challenge with an Egyptian H5N1 virus belonged to the 2.2.1/C group [31]. Therefore, we assume that improper administration, mishandling and inappropriate storage of the vaccine [7] or suppression of the immune system (i.e.: due to chicken anemia virus infection or ingestion of mycotoxins) [32] could be responsible for such weak immune response and subsequently lack of protection. Moreover, the ability of inactivated H5N1 vaccine to evoke effective immune response in Hi-Sex brown layers remains to be investigated. Taken together, vaccination with regularly updated H5N1 vaccines to protect poultry against the evolving H5N1 virus in Egypt is highly recommended.

The high level of genetic identity of A/chicken/Egypt/0987-NLQP/2009 to viruses in LBM markets and backyard birds suggests a direct transmission link which is not uncommon scenario in Egypt. On the other hand, close genetic relationship between A/chicken/Egypt/0987-NLQP/2009 and H5N1 virus isolated from 32 months old boy from the same village could be explained by existence of a common source of infection; most probably backyard birds and/or LBM [7,9]. However, another possible source of infection could be human-to-chicken transmission which instantly could neither be excluded nor confirmed. Subclinical infections of human with H5N1 virus probably due to poultry-to-human, limited human-to-human transmission or environmental source(s) have been reported in China, Cambodia, Vietnam, Thailand and Turkey [33-37]. It is worth pointing out that subclinical infections of apparently healthy pigs and donkeys with HPAIV H5N1 have been reported in Egypt [8,10]. Unfortunately there is a paucity of information on subclinical spread among people in Egypt, particularly those in close contact to infected backyard birds or shared in culling of infected commercial farms. However, since late 2008, symptomless cases infected with mild virulent H5N1 in Egypt have raised concern that the virus might be adapted to Egyptians without getting sick [38].

It is well known that affinity of H5N1 virus to avian-type α2-3 and mammalian-like α2-6 linked sialic acid receptors seems to be governed by a number of residues in the HA protein including serine at position 129 which is a part of the receptor binding domain (RBD) [39]. It has been found that the Egyptian H5N1 viruses had a potential to use mammalian receptors resembling seasonal H1N1 virus [40]**.** Deletion of RBD 129S found in A/chicken/Egypt/0987-NLQP/2009 (Figure 2) existed also in all recent isolates of human and backyard origins in Egypt but had neither been reported from the parent GsGd/96 virus [27] nor from Index/2006, the virus originally introduced into Egypt in 2006 . Intriguingly, virus isolated from donkeys belonged to the same 2.2.1/C sublineage (Figure 1). Recently, deletion of RBD 129S combined with I151T in this unique Egyptian genetic group increased affinity of the Egyptian viruses to mammalian receptors and retained its avian receptor specificity [15]. Mutation in this residue was associated with a less virulent H5N1 phenotype causing milder or asymptomatic courses of infection and increased transmissibility in mice [41]. Interestingly, other significant substitutions such as Q192R, G222L and Q224S associated with adjustment of the virus from avian to mammalian receptors [42] were not observed in any Egyptian H5 gene sequences, including A/chicken/Egypt/0987-NLQP/2009 [13].

It is well known that N-linked glycosylation of the HA of influenza A viruses can affect receptor binding preferences or mask antigenic regions [41]. A/chicken/Egypt/0987-NLQP/2009 like other recent Egyptian H5N1 viruses of human and backyard origin, lost the potential glycosylation site at residue 154 (near the RBD) [13]. This phenotype was associated with (1) increase the affinity of H5 viruses to α2-6 residues [43], (2) increased transmissibility in guinea pigs and decreased fatality and systemic spread in mice [44]. Taken together, these clues could support the possible scenario of human-to-poultry transmission in Egypt. Therefore, infected human (farm workers, dealers, visitors, etc.) in Egypt should be considered a possible source of infection not only mechanical but also as a biological vector. Moreover, targeted surveillance to identify subclinical infection of human in Egypt should be taken in consideration to avoid a sudden emergence of pandemic virus. On the other hand, residue A46 is located in the NA stalk region while the others are positioned at the surface of the NA monomer (Figure 2). None of NA mutations has a known biological function except 319S which is a part of an immunogenic epitope (C) (56). A similar deletion in human H1N1 virus brought with it compensatory changes in the NA to facilitate viral entry and release and a similar mechanism may be acting on the 2.2.1/C viruses of human origin in Egypt [45]. Recently, isolation of H9N2 has been isolated from poultry in Egypt and possible reassortment is expected [46-48].

In conclusion, circulation of HPAIV H5N1 in vaccinated birds continues to devastate the poultry industry in Egypt. Birds in backyards and LBM remain the main potential source of H5N1 infection to both commercial poultry and humans in Egypt. Targeted surveillance to elucidate the spread of HPAIV H5N1 among commercial poultry workers and/or householders should be considered.

## Abbreviations

AIV: Avian influenza virus; D: Aspartic acid; EID: Egg-infective dose; HA: Hemagglutinin; HI: Hemagglutinin inhibition; HPAI: Highly pathogenic avian influenza; I: Isoleucine; G: Glycine; LBM: Live birds markets; N: Asparagine; NLQP: National laboratory for quality control on poultry production; OIE: World organization of animal health; RBD: Receptor binding domain; RT-qPCR: Real-time reverse transcription polymerase chain reaction; SPF: Specific pathogen free; S: Serine; T: Threonine.

## Competing interests

The authors declare that they have no competing interests.

## Authors’ contributions

EFE carried out sample collection and examination and helped to draft the manuscript. AA carried out the sequence of the isolated virus. WHK participated in virus isolation and/or serological examination. EMA did sequence and phylogenetic analyses and helped to draft the manuscript. MMA and HMH conceived and coordinated the study and helped to draft the manuscript. All authors read and approved the final manuscript.
